# Turning over New Leaves

**Published:** 1889-05-25

**Authors:** 


### Turning Over New Leaves.

(Books for Review should be sent to The Editor, The Lodge, Porchester Square, W.)

### FOR THE RIGHT.*

A book which comes to us franked by George MacDonald, and which has been favourably noticed by Mr. Gladstone, is nearly sure to be widely read in England, and as we finished the last chapter of "For the Right," we laid the volume down with the reflection that hardly any circulation could be too great for the merits shown in it. Plot, one might say, there is next to none, and the interest of the book centres around the life of one man, named Tarus Barabola. We get once more presented to us, in terribly graphic form, the old, old problem—"Man's destiny here below." The author of the Book of Job clothed the same problems in Eastern imagery, and decorated it with a literary skill never since equalled; Goethe burdened his tragic muse with it; Victor Hugo gave us his idea of it in the apotheosis of Jean Valjean, the finest, or at least the most perfect, character in modern fiction. Fronzos treats the problem very differently from either Goethe or Hugo. His Taras is wretchedly poor, has, moreover, a stain on his birth, and yet, by the mere innate force of goodness, and following the guiding star of his burning love of justice, he triumphs over all his difficulties, leads an ideal life for a time, thinks himself bound to forsake it owing to a miscarriage of the law—of that justice which he worships—ends by being unjust himself, and expiates his crime by surrendering his life.

The scene is laid in the wilds of the Carpathian mountains, and among a wild people. "Life there," says Fronzos, "on the whole, is regulated after the ways of the lowlands; but the people themselves approach the Huzul type, a peculiar race, inhabiting the mountains, and which, on account of the common language, is generally classed with the Ruthens, but being of a different origin, and of different conditions of life, is distinct from them, as in appearance, so in habit and in character. The Huzul is a hybrid, uniting the Sclavonic blood of the Ruthen with the Mongolian blood of the Uzen, his speech betraying the former, while his name testifies to the latter. So, also, does the defiant dauntlessness of his bearing, hidden beneath an appearance of proud restraint, but apt to burst out suddenly, like a hot spring, through the covering snow." We quote this passage as a sample of the author's style, and of his translator's power to render the German into English. It is, too, refreshing to find a writer of fiction who has grasped even a part of the significance of the great question of "race," a question which really dominates every other, and by which every other must be judged. All through the pages of "For the Right" we feel that it is not Saxon, and we should be surprised to learn that Mr. Fronzos is German in any other sense than that he speaks the German language. The strong, practical common sense of the Saxon would have had no hope of regenerating a community where half of the people were ready to perjure themselves at the bidding of a great man's greatman. It needs the Sclavonic or Celtic idealism to undertake such a task.

The book seems to be very well done into English. It does not read painfully like a translation. We think, however, the "and which" error might have been steered clear of.

* "For the Right." By Karl Emil Fronzos. Given in English by Julie Sutter James Clarke and Co. Pp. 531.;

